# Association between maternal insecticide use and otitis media in one-year-old children in the Japan Environment and Children’s Study

**DOI:** 10.1038/s41598-022-05433-2

**Published:** 2022-01-25

**Authors:** Takeshi Utsunomiya, Naoko Taniguchi, Yohei Taniguchi, Tetsuro Fujino, Yasuhiko Tanaka, Hideki Hasunuma, Masumi Okuda, Masayuki Shima, Yasuhiro Takeshima, The Japan Environment, and Children’s Study Group, Michihiro Kamijima, Shin Yamazaki, Yukihiro Ohya, Reiko Kishi, Nobuo Yaegashi, Koichi Hashimoto, Chisato Mori, Shuichi Ito, Zentaro Yamagata, Hidekuni Inadera, Takeo Nakayama, Hiroyasu Iso, Masayuki Shima, Youichi Kurozawa, Narufumi Suganuma, Koichi Kusuhara, Takahiko Katoh

**Affiliations:** 1 Department of Pediatrics, Hyogo College of Medicine, 1-1 Mukogawa Nishinomiya, Hyogo, 663-8501 Japan; 2 Hyogo Regional Center for the Japan Environment and Children’s Study, Nishinomiya, Japan; 3 Department of Public Health, Hyogo College of Medicine, Nishinomiya, Japan; 4 Nagoya City University, Nagoya, Japan; 5 National Institute for Environmental Studies, Tsukuba, Japan; 6 National Center for Child Health and Development, Tokyo, Japan; 7 Hokkaido University, Sapporo, Japan; 8 Tohoku University, Sendai, Japan; 9 Fukushima Medical University, Fukushima, Japan; 10 Chiba University, Chiba, Japan; 11 Yokohama City University, Yokohama, Japan; 12 University of Yamanashi, Chuo, Japan; 13 University of Toyama, Toyama, Japan; 14 Kyoto University, Kyoto, Japan; 15 Osaka University, Suita, Japan; 16 Tottori University, Yonago, Japan; 17 Kochi University, Nankoku, Japan; 18 University of Occupational and Environmental Health, Kitakyushu, Japan; 19 Kumamoto University, Kumamoto, Japan

**Keywords:** Risk factors, Infectious diseases, Paediatrics

## Abstract

Otitis media (OM) is common among young children and is related to hearing loss. We investigated the association between maternal insecticide use, from conception to the first and second/third trimesters, and OM events in children in the first year of age. Data from Japan Environment and Children's Study were used in this prospective cohort study. Characteristics of patients with and without history of OM during the first year of age were compared. The association between history of OM in the first year and insecticide use was evaluated using logistic regression analysis. The study enrolled 98,255 infants. There was no significant difference in the frequency of insecticide use between groups. Insecticide use of more than once a week from conception to the first trimester significantly increased the occurrence of OM in children in the first year (odds ratio [OR] = 1.30, 95% confidence interval [CI] 1.01–1.67). The association between OM in the first year and insecticide use from conception to the first trimester was only significant in the group without daycare attendance (OR 1.76, 95% CI 1.30–2.38). Maternal insecticide use more than once a week from conception to the first trimester significantly increased OM risk in offspring without daycare attendance.

## Introduction

Otitis media (OM) is a spectrum of diseases, including acute otitis media (AOM), OM with effusion, and chronic suppurative OM^1^. OM is a common disease among children worldwide. The average AOM incidence was 10.8 new episodes per 100 people per year^2^. *Streptococcus pneumoniae*, non-typable *Haemophilus influenzae* and *Moraxella catarrhalis* are three major bacterial pathogens which cause OM^3^. Many host and environmental factors can increase the risk of OM. Factors include young age^4^, male sex^5^, maternal age < 20 years^5^, non-Hispanic white ethnicity^5^, family history of recurrent OM^6^, exposure to tobacco smoke^6^, having an older sibling^7^, the use of a pacifier^8^, trisomy 21^9^, gestational week^10^, birth weight^11^, maternal occupation^12^, and daycare attendance^4,7,13^. Attending daycare and having siblings (generally older) were consistent risk factors for OM with effusion^14–16^. Preventive factors were exclusive breastfeeding for the first 6 months^17^.

Pyrethroids and organochlorine are two major insecticides. There were reports of insecticide exposure during pregnancy influencing the offspring. Perinatal exposure to pyrethroids was associated with attention deficit hyperactivity disorder at 2–4 years of age^18^. Maternal prenatal pyrethroid metabolite concentrations were not consistently associated with children’s cognitive scores^19^. An adverse association of prenatal exposure to pyrethroids as measured by urinary metabolites with birth weight was also reported^20^. Moreover, maternal exposure to pyrethroid insecticide during pregnancy strangely had a positive effect on infant development^21^. Regarding the association between OM history and insecticide exposure, prenatal organochlorine exposure could be a risk factor for AOM in Inuit infants^22^.

We aimed to investigate the association between children’s OM history at the age of one and the frequency of maternal insecticide use both from conception to the first and second/third trimesters.

## Results

**Figure 1:**
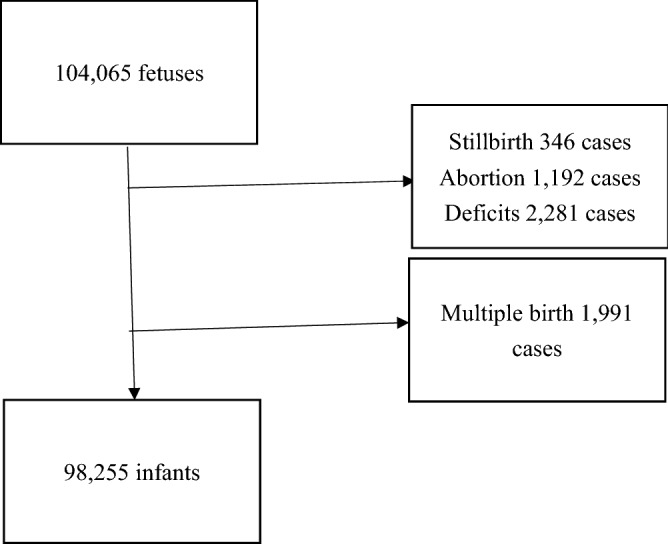
Enrollment chart.

We recruited 104,065 fetuses and excluded 346 stillbirth cases, 1192 abortion cases, 2281 missing values, and 1991 multiple births. Finally, we included 98,255 infants in the present study (Fig. 1).

We compared the frequency of maternal insecticide use both after conception to the first and second/third trimesters between the OM group and no OM group (Table 1).

**Table 1:** Comparison of maternal insecticide use frequency between the otitis media and no otitis media groups.

	OM (-)	OM ( +)
**Usage of insecticide by the mother from conception to the first trimester (n = 79,098)**		
Never (%)	66,054 (94)	8,505 (94)
1 to 3 times per month (%)	3,365 (5)	428 (5)
More than once a week (%)	646 (1)	100 (1)
**Usage of insecticide by the mother from conception to the second/third trimester (n = 84,544)**		
Never (%)	68,612 (92)	8,818 (91)
1 to 3 times per month (%)	5,401 (7)	726 (8)
More than once a week (%)	875 (1)	112 (1)

*OM* otitis media.

There were no significant differences in other patient characteristics, including sex, maternal age, birth weight, gestational age, living with siblings at 6 months, daycare attendance at the age of 1 year, maternal history of chronic otitis media, maternal and paternal smoking history, exposure to secondhand smoke, infertility treatment, family member’s smoking status, and maternal occupational categories between the otitis media and no otitis media groups in the first year in children.

Whether children were affected by OM during the first year was associated with that mothers used insecticide more than once a week from becoming pregnant to the first trimester (odds ratio [OR] = 1.30, 95% confidence interval [CI] 1.01–1.67). Whether children were affected with OM during the first year was not associated with that mothers used insecticide from becoming pregnant to the second/third trimester (Table 2).

**Table 2:** Association between history of otitis media in the first year in children and maternal insecticide use from conception to the first (upper) and the second/third (lower) trimesters.

	Adjusted OR	95% C.I	p value
**Usage of insecticide by the mother from conception to the first trimester (n = 57,886)**			
Never	Reference		
1 to 3 times per month	0.97	0.86–1.10	0.62
More than once a week	1.30	1.01–1.67	0.04
**Usage of insecticide by the mother from conception to the second/third trimester (n = 61,580)**			
Never	Reference		
1 to 3 times per month	1.06	0.97–1.17	0.22
More than once a week	1.01	0.80–1.27	0.94

*OR* odds ratio, *95% C.I.* 95% confidence interval.

These analyses were adjusted for sex, exclusive breastfeeding for the first 6 months, maternal age, gestational age, birth weight, living with siblings at 6 months, daycare attendance, maternal history of chronic otitis media, Hib vaccination, pneumococcal vaccination, maternal and paternal smoking history, exposure to secondhand smoke, diagnosis of trisomy 21, fertility treatment, family member’s smoking status, and maternal occupational category.

The result of estimation using multiple imputations confirmed the outcome of the logistic regression analysis (Table 3). In subgroup analysis, whether children were affected with OM for the first year was significantly associated with that mothers used insecticide more than once a week from conception to the first trimester in sub-group of no attendance to daycare (OR 1.76, 95% CI 1.30–2.38) (Table 4).

**Table 3:** Estimation by multiple imputation of the association between history of otitis media in the first year in children and maternal insecticide use from conception to the first (upper) and the second/third (lower) trimesters.

	Adjusted OR	95% C.I	p value
**Usage of insecticide by the mother from conception to the first trimester (n = 57,886)**			
Never	Reference		
1 to 3 times per month	1.01	0.91–1.12	0.89
More than once a week	1.29	1.03–1.62	0.03
**Usage of insecticide by the mother from conception to the second/third trimester (n = 61,580)**			
Never (%)	Reference		
1 to 3 times per month (%)	1.07	0.98–1.16	0.13
More than once a week (%)	0.95	0.78–1.16	0.62

*OR* odds ratio, *95% C.I.* 95% confidence interval.

These analyses were adjusted for sex, exclusive breastfeeding for the first 6 months, maternal age, gestational age, birth weight, living with siblings at 6 months, daycare attendance, maternal history of chronic otitis media, Hib vaccination, pneumococcal vaccination, maternal and paternal smoking history, exposure to secondhand smoke, diagnosis of trisomy 21, fertility treatment, family member’s smoking status, and maternal occupational category.

**Table 4:** Association between the history of otitis media in the first year in children and maternal insecticide use from conception to the first trimester in subgroup analysis.

Subgroup	Adjusted OR	95% C.I	Adjusted OR	95% C.I
	No sibling at 6 months (n = 25,054)		With sibling at 6 months (n = 32,821)	
**Usage of insecticide by the mother from conception to the first trimester**				
Never	Reference		Reference	
1 to 3 times per month	0.80	0.61–1.05	1.03	0.89–1.18
More than once a week	1.58	0.97–2.58	1.23	0.92–1.65

Subgroup	Day care attendance (n = 15,757)		No day care attendance (n = 42,125)	
**Usage of insecticide by the mother from conception to the first trimester**				
Never	Reference		Reference	
1 to 3 times per month	0.89	0.74–1.07	1.04	0.88–1.23
More than once a week	0.79	0.51–1.23	1.76	1.30–2.38

Subgroup	Mother has no history of Chronic OM (n = 56,986)		Mother has history of Chronic OM (n = 888)	
Usage of insecticide by the mother from conception to the first trimester				
Never	Reference		Reference	
1 to 3 times per month	0.97	0.86–1.10	0.81	0.31–2.09
More than once a week	1.29	1.00–1.67	1.83	0.37–8.99

*OR* odds ratio, *95% C.I.* 95% confidence interval, *OM* otitis media.

There were three subgroups, including living with siblings at 6 months, daycare attendance, and maternal history of chronic otitis media. These analyses were adjusted for sex, exclusive breastfeeding for the first 6 months, maternal age, gestational age, birth weight, living with siblings at 6 months, daycare attendance, maternal history of chronic otitis media, Hib vaccination, pneumococcal vaccination, maternal and paternal smoking history, exposure to secondhand smoke, diagnosis of trisomy 21, fertility treatment, family member’s smoking, and maternal occupational category.

There was no significant association between whether children were affected with OM during the first year and that mothers used insecticide from conception to the second/third trimester in all subgroups (Table 5).

**Table 5:** Association between the history of otitis media in the first year in children and maternal insecticide use from conception to the second/third trimester in subgroup analysis.

Subgroup	Adjusted OR	95% C.I	Adjusted OR	95% C.I
	No sibling at 6 months (n = 26,389)		With sibling at 6 months (n = 35,178)	
**Usage of insecticide by the mother from conception to the second/third trimester**				
Never	Reference		Reference	
1 to 3 times per month	1.16	0.95–1.41	1.04	0.93–1.16
More than once a week	1.48	0.93–2.37	0.91	0.70–1.18

Subgroup	Day care attendance (n = 16,603)		No day care attendance (n = 44,973)	
**Usage of insecticide by the mother from conception to the second/third trimester**				
Never	Reference		Reference	
1 to 3 times per month	1.11	0.97–1.28	1.01	0.89–1.16
More than once a week	0.88	0.63–1.24	1.12	0.83–1.52

Subgroup	Mother has no history of Chronic OM (n = 60,613)		Mother has history of Chronic OM (n = 955)	
**Usage of insecticide by the mother from conception to the second/third trimesters**				
Never	Reference		Reference	
1 to 3 times per month	1.07	0.97–1.18	0.83	0.44–1.59
More than once a week	1.01	0.80–1.28	0.95	0.19–4.72

*OR* odds ratio, *95% C.I.* 95% confidence interval, *OM* otitis media.

There were three subgroups, including living with siblings at 6 months, daycare attendance, and maternal history of chronic otitis media. These analyses were adjusted for sex, exclusive breastfeeding for the first 6 months, maternal age, gestational age, birth weight, living with siblings at 6 months, daycare attendance, maternal history of chronic otitis media, Hib vaccination, pneumococcal vaccination, maternal and paternal smoking history, exposure to secondhand smoke, diagnosis of trisomy 21, fertility treatment, family member’s smoking and maternal occupational category.

## Discussion

In the present study, we investigated the correlation between OM history in the first year in children and maternal insecticide use from conception to the first and second/third trimesters using multivariate regression analysis.

Previous studies have shown no association between prenatal domestic insecticide exposure and OM episode during early childhood^23^. The population was 3,421 women before 19th week of gestation in a region in France. The women occupationally exposed to pesticide were excluded. Outcome was OM episode in the first two years in children and was collected with questionnaire from mothers. Exposure included eight different types of home pesticides for plants, fleas and ticks, insects, rodents, and house frame treatment. The number of times domestic insecticide was used was only 1,426. Exposure scores were rated from 1 (low exposure) to 3 (high exposure). The differences between the previous study and ours include population size and exposure period, outcome assessment period, and the details of pesticide use. Our population was much larger and covered a wider geographical location compared with the previous study. The period of maternal pesticide exposure was until 19^th^ week of gestation and the second/third trimester. We focused not only on this period, but from conception to the first trimester as well; therefore, we can reveal the association between OM for a more limited period and maternal pesticide use for a more important gestational period in a larger population compared with the previous study. Maternal insecticide use from conception to the first trimester at the frequency of more than once a week may be avoided if mothers do not intend to have their children attend the daycare at the age of 1 year.

The mechanism of relation between use of insecticide and OM may be caused by deteriorated immune function. Previous studies showed the level of IgA were lower in breast-fed infants from prenatally organochlorine-exposed mothers rather than in bottle-fed infants both at seven and at 12 months^22^. In other study, the strongest dose-dependent association between prenatal exposure to organochlorine and ear infection was shown^24^. Perinatal accumulation of organochlorine to mother may negatively affect the immune system in infants.

In our analysis presenting at Tables 2 and 3, living with older siblings, the history of maternal chronic OM, and daycare attendance increased the incidence of OM. Thus, our results were consistent with the findings of the previous studies^5,25,26^.

The outcome of the present study may contribute to decreased otitis media prevalence and incidence in children for the first year when pregnant woman are advised to avoid using insecticide. As the cost of OM treatment in the United States is estimated at between three to four billion dollars^27^, decreasing OM results in huge financial savings. Approximately 17 percent of children have three or more episodes during a 6 months period^27^. Preventing OM decreases the burden that is placed on parents who have to visit clinics and take time off work.

One limitation of this study was the lack of detailed information on the type and amount of mothers’ insecticide exposure. It is important which insecticide and how much dose mother used between conception both to the first trimester and second/third trimesters.

Maternal insecticide use before conception may also be a limitation because of recall bias. Some mothers may misunderstand the timing of insecticide use or may misunderstand other products, such as pesticides as an insecticide.

Another limitation is that we cannot exclude the possibility of unknown confounding factors that influence the frequency of maternal insecticide use.

In conclusion, despite these limitations, we revealed that maternal insecticide use more than once a week in the group without daycare attendance at the age of 1 year was associated with the history of OM in the first year of age of children. Thus, we need further investigation into how maternal insecticide use influences OM in their children.

## Methods

The STROBE checklist was followed in this study.

### Study design and participants

We used data from the Japan Environment and Children's Study (JECS) in this prospective cohort study. The JECS is a national birth cohort study investigating the environmental factors that potentially affect the health and development of children in Japan^28^. The details of the JECS have been previously described^29^. All participants were recruited between January 2011 and March 2014. The present study used the "jecs-an-20180131" dataset, which was released in March 2018. Of these, we excluded stillbirth, abortion, missing values, and multiple birth patients.

### Data collection

Data regarding first year OM history, breastfeeding for the first 6 months, daycare use, pneumococcal, and Hib vaccine were collected from a parental self-reported questionnaire. Data regarding live births or stillbirths, singleton or multiple births, sex, gestational weeks, birth weight, maternal age, and whether the child was diagnosed with trisomy 21 were transcribed from medical records by physicians, midwives/nurses, and research co-ordinators.

The primary outcome was obtained by “C1y questionnaire”, which asked caregivers when their children were at the age of 1 “Has your child been diagnosed with OM by a doctor?” The outcome variable was binomial: no diagnosis with OM as 0 and diagnosis with OM as 1. We did not distinguish between acute otitis media and otitis media with effusion in this questionnaire. Further, there was no data how OM was treated in this questionnaire.

The exposure factors were maternal occupational use of insecticide more than half a day from conception to the first and second/third trimesters. The question was, “What was the frequency of occupational use of insecticide for more than half a day during pregnancy?”. We obtained this information from the questionnaire to mother” M-T1 questionnaire”, filled in the first trimester, and “M-T2 questionnaire”, filled in the second/third trimester^30^. We classified these variables as follows: No as 1, 1–3 times per month as 2, and 1–6 times a week and everyday as 3.

We selected the covariates below because they were known risk factors of OM as described in the introduction section. Although maternal infertility treatment was not reported to be associated with OM; primary ciliary dyskinesia, which causes both chronic OM and infertility, is inherited^31^. Therefore, we selected maternal infertility treatment as covariate. Other covariates included sex, gestational weeks, birth weight, exclusive breastfeeding for the first 6 months, living with older siblings at 6 months, nursery attendance at the age of one, maternal history of chronic OM, maternal age, a history of pneumococcal vaccine, and Hib vaccine, whether the child was diagnosed with trisomy 21 or not, and history of fertility treatment. We also selected smoking around children, such as paternal and maternal smoking habits, maternal exposure to secondhand smoking, and household smoking at the age of 1 month. We focused on maternal occupation, especially farmers. However, the number of farmers was very small. Instead, we used major occupational categories. We recorded the number of mothers in every major maternal occupational category.

We classified the binomial variables as follows: sex of the child: male as 1, female as 2; living with siblings at 6 months, daycare use at a year old, maternal history of chronic OM, history of pneumococcal vaccine and Hib vaccine, children with trisomy 21, history of fertility treatment: No as 0, yes as 1.

Furthermore, we classified categorical variables as follows: gestational weeks^32,33^: 37 to 41 weeks as 1, < 37 weeks as 2, ≥ 42 weeks as 3; the birth weight of child: 2.5 -4 kg as 1, < 2.5 kg as 2, ≥ 4 kg as 3; maternal age: 30–34 years as 1, 25–29 years as 2, < 25 years as 3, and ≥ 35 years as 4; paternal and maternal smoking habits: never as 1, "previously did, but quit before realizing current pregnancy" as 2, "previously did, but quit after realizing current pregnancy" as 3, and currently smoking as 4; the frequency of maternal exposure to secondhand smoking: never as 1, more than once a week as 2; household smoking at the age of 1 month: no one as 1, someone smoking at a place far from the baby as 2, smoking at a location near the baby as 3. Maternal occupational categories: full-time homemaker as 1, professional and technicians as 2, clerical support workers as 3, service workers as 4, the others as 5 because, of major occupational categories, the biggest category was full-time homemakers (n = 23,890), followed by professional and technicians (n = 18,758), clerical support workers (n = 14,633), service workers (n = 12,901), and the others (n = 14,308). For all variables, we set the minimum category number as a reference control.

### Statistical analysis

All statistical analyses were performed using Stata version 15 software (StataCorp, College Station, TX, USA). First, we compared patient characteristics between the OM history and non-OM history groups for the first year in children. Second, we analyzed the relationship between whether children were affected with OM during the first year and maternal insecticide use, both from conception to the first trimester and from conception to the second/third trimesters, using logistic regression analysis. Third, we performed sensitivity analysis by the multiple imputation method since there were many missing values. Finally, we performed sub-group analysis of the relationship between whether children were affected with OM for the first year and maternal insecticide use both from conception to the first trimester, and from conception to the second/third trimesters using logistic regression analysis in three subgroups. These groups included whether children had a sibling living with them when they were 6 months, whether children attended daycare at the age of one, and whether the mother had a history of chronic OM. Significance was defined as p < 0.05.

### Ethical considerations

The JECS protocol was reviewed and approved by the Ministry of the Environment’s Institutional Review Board on Epidemiological Studies and the Ethics Committees of all participating institutions: the National Institute for Environmental Studies that leads the JECS, the National Center for Child Health and Development, Hokkaido University, Sapporo Medical University, Asahikawa Medical University, Japanese Red Cross Hokkaido College of Nursing, Tohoku University, Fukushima Medical University, Chiba University, Yokohama City University, University of Yamanashi, Shinshu University, University of Toyama, Nagoya City University, Kyoto University, Doshisha University, Osaka University, Osaka Medical Center Research Institute for Maternal and Child Health, Hyogo College of Medicine, Tottori University, Kochi University, University of Occupational and Environmental Health, Kyushu University, Kumamoto University, University of Miyazaki, University of Ryukyu (Ethical number: No.100910001). We confirmed that all research was performed in accordance with relevant guidelines/regulations. We obtained written informed consent from all participants before participation in the present study.

## Data Availability

Data are unsuitable for public deposition due to ethical restrictions and the legal framework of Japan. It is prohibited by the Act on the Protection of Personal Information (Act No. 57 of May 30, 2003, amendment on September 9, 2015) to publicly deposit data containing personal information. Ethical Guidelines for Medical and Health Research Involving Human Subjects enforced by the Japan Ministry of Education, Culture, Sports, Science and Technology and the Ministry of Health, Labour and Welfare also restrict the open sharing of epidemiologic data. All inquiries regarding access to data should be sent to jecs-en@nies.go.jp. The person responsible for handling questions sent to this email address is Dr. Shoji F. Nakayama, JECS Program Office, National Institute for Environmental Studies.
